# Association between undiagnosed obstructive sleep apnea and severe course of COVID-19: a prospective observational study

**DOI:** 10.1007/s11325-023-02855-8

**Published:** 2023-07-07

**Authors:** Natalia Celejewska-Wójcik, Kamil Polok, Karolina Górka, Tomasz Stachura, Aleksander Kania, Paweł Nastałek, Sabina Lichołai, Weronika Zastrzeżyńska, Marek Przybyszowski, Krzysztof Sładek

**Affiliations:** 1 Department of Pulmonology and Allergology, University Hospital, Kraków, Poland; 2 2nd Department of Internal Medicine, Jagiellonian University Medical College, Kraków, Poland; 3 Centre for Intensive Care and Perioperative Medicine, Jagiellonian University Medical College, Kraków, Poland; 4 Division of Molecular Biology and Clinical Genetics, Department of Medicine, Jagiellonian University Medical College, Kraków, Poland

**Keywords:** Obstructive sleep apnea, COVID-19, Respiratory failure, Non-invasive ventilation

## Abstract

**Purpose:**

Obstructive sleep apnea (OSA) is associated with many long-term health consequences. We hypothesized that previously unrecognized and untreated OSA may be associated with more severe respiratory failure in hospitalized patients with COVID-19.

**Methods:**

Patients hospitalized in the Pulmonology Department with confirmed COVID-19, University Hospital in Kraków, Poland, between September 2020 and April 2021 were enrolled**.** OSA screening questionnaires including Epworth Sleepiness Scale (ESS), STOP-BANG, Berlin questionaire (BQ), OSA-50, and No-SAS were completed. Polygraphy was performed after > 24 h without requirement for supplemental oxygen.

**Results:**

Of 125 patients with median age of 61.0 years, 71% of whom were male. OSA was diagnosed in 103 patients (82%) and was categorized as mild, moderate, and severe in 41 (33%), 30 (24%), and 32 (26%), respectively. Advanced respiratory support was introduced in 85 patients (68%), and 8 (7%) patients eventually required intubation. Multivariable analysis revealed that increased risk of requirement for advanced respiratory support was associated with higher respiratory event index (OR 1.03, 95%CI 1.00 to 1.07), oxygen desaturation index (OR 1.05, 95%CI 1.02 to 1.10), and hypoxic burden (1.02 95% CI 1.00 to 1.03) and lower minimal SpO_2_ (OR 0.89, 95%CI 0.81 to 0.98), but not with results of OSA screening tools like BQ score (OR 0.66, 95%CI 0.38 to 1.16), STOP-BANG score (OR 0.73, 95%CI 0.51 to 1.01), NoSAS score (OR 1.01, 95%CI 0.87 to 1.18), or OSA50 score (OR 0.84, 95%CI 0.70 to 1.01).

**Conclusion:**

Previously undiagnosed OSA was common among hospitalized patients who survived the acute phase of COVID-19. The degree of OSA was associated with the severity of respiratory failure.

**Supplementary Information:**

The online version contains supplementary material available at 10.1007/s11325-023-02855-8.

## Introduction

Coronavirus disease 2019 (COVID-19) affects predominantly the respiratory system, with dyspnea being the most frequent symptom presaging progressive life-threating respiratory failure [1]. In studies conducted in the early phases of the pandemic on large cohorts of symptomatic COVID-19 patients, approximately 15% of patients had severe disease requiring hospitalization, and 5% became critically ill. Nearly half of the patients admitted to the intensive care unit (ICU) did not survive the disease [2]. Older age, male sex, and comorbidities such as obesity, hypertension, and diabetes have been identified as risk factors for worse outcomes in SARS-CoV-2 infection [3, 4]. High flow nasal oxygen (HFNO) and positive airway pressure (PAP) are widely accepted treatment modalities for severe respiratory failure in COVID-19 pneumonia [5–7]. The need for advanced respiratory support in COVID-19 pneumonia was associated with worse outcomes [8–10].

Obstructive sleep apnea (OSA) is the most common sleep-related breathing disorder, yet it is believed to be highly underdiagnosed. A study conducted in the USA in the 1990s suggested that 93% of women and 82% of men with moderate to severe OSA might remain undiagnosed [11]. Recurrent episodes of complete or partial upper airway collapse, termed as apneas and hypopneas, lead to serious physiological consequences: nocturnal saturation disturbances, disruption of sleep structure, and activation of the autonomic nervous system [12]. Male sex, older age, family history, excessive body weight with central body fat distribution, large neck circumference, craniofacial and upper airway abnormalities are well-established risk factors for OSA [13].

A relationship between OSA and risk of a more severe course of COVID-19 has mostly been described in registry-based studies so far. It was shown that the diagnosis of OSA was associated with higher risk of developing respiratory failure, requirement for hospitalization, and COVID-19 death [13–15]. This relationship is probably multifactorial. First, conditions coexisting with OSA such as hypertension, coronary artery disease, stroke, congestive heart failure, diabetes, and metabolic syndrome [13] largely overlap with the above-mentioned risk factors for severe COVID-19. Second, endothelial dysfunction, inflammation, and oxidative stress associated with OSA add to the damage of the lungs caused by SARS-CoV-2 infection [14, 15].

We hypothesized that previously unrecognized and untreated OSA may be associated with more severe respiratory failure in hospitalized patients with COVID-19. The aim of this study was to evaluate the relationship between the degree of undiagnosed sleep-related breathing disorders and requirement for more advanced respiratory support in patients hospitalized due to COVID-19.

## Patients and methods

### Study design

The recruitment of patients for this prospective observational study took place between September 2020 and April 2021 in the Department of Pulmonology and Allergology, University Hospital in Kraków, Poland. Patients hospitalized with SARS-CoV-2 who survived the acute phase of the disease were tested for previously undiagnosed sleep-related breathing disorders. The study protocol was in line with the Declaration of Helsinki and was approved by the Ethics Committee of Jagiellonian University Medical College, Kraków, Poland (KBET 1072.6120.145.2020, Chairperson Prof. Piotr Thor) on May 28, 2020. All study subjects provided a written informed consent.

### Patients and data collection

Consecutive patients admitted to the hospital with COVID-19 confirmed using a RT-PCR test of a nasal swab were enrolled. Patients who died during hospitalization or were previously diagnosed with OSA were excluded from the study. Demographic and clinical characteristics of the study participants were obtained based on the available medical records and medical interview with the patient or the relatives. OSA screening questionnaires including Epworth Sleepiness Scale (ESS), STOP-BANG, Berlin questionaire (BQ), OSA-50, and No-SAS were acquired [16–20]. Polygraphy was performed after > 24 h without requirement for supplemental oxygen.

### OSA diagnosis and treatment

The diagnosis of OSA was established through four-channel polygraphy, including measurements of airflow, thoraco-abdominal movements, blood oxygenation, and snoring (Alice NightOne, Philips Respironics), according to AASM guidelines [21]. The respiratory event index (REI) was defined as the number of apneas or hypopneas recorded during the study per hour of device recording time. REI cutoff values indicated mild (REI 5 up to 15/h), moderate (REI 15 up to 30/h), and severe OSA (REI ≥ 30/h), respectively. Hypoxic burden (HB), an additional measure of nocturnal hypoxemia, was determined by measuring sleep apnea-associated area under the desaturation curve for total sleep time [22]. In all patients with indications for CPAP therapy, auto-CPAP device (Dream Station CPAP Pro, Philips Respironics) was used, and therapeutic pressure was titrated.

### Scales

In the Epworth Sleepiness Scale questionnaire, respondents are asked to rate their usual risks of nodding off or falling asleep while engaged in eight different activities. Scores ranging from 11 to 24 indicate increasing levels of excessive daytime sleepiness [16]. Eight dichotomous questions in the STOP-BANG questionnaire relate to clinical symptoms of sleep apnea, including snoring, fatigue, witnessed apnea, high blood pressure, BMI, age, neck circumference, and male gender. Patients with scores of 5 to 8 are classified as high risk for moderate to severe OSA [18]. The Berlin questionnaire consists of 10 questions in three categories related to the presence and severity of snoring, frequency of daytime sleepiness, and the presence of obesity or hypertension. Positive scores in at least two out of three categories indicates patients to be at high risk of OSA [17]. The No-SAS screening tool assesses 5 parameters: neck circumference, obesity, snoring, age, and sex. The patient presents with a high probability of OSA when scores of 8 points or higher [20]. The OSA-50 screening questionnaire takes into account waist circumference, history of snoring, witnessed apneas, and age. Scores of 5 and more have been found to identify patients with increased risk of moderate to severe OSA [19].

### Outcomes

The primary outcome was the requirement for advanced respiratory support defined as high-flow nasal oxygen therapy (HFNO) or non-invasive ventilation (NIV). Other described outcomes included intubation rate and length of ICU stay and hospitalization.

### Statistical analysis

Categorical variables were presented as numbers (%) and compared using chi-square test or Fisher’s exact test as appropriate. Continuous variables were presented as mean (standard deviation) or median (interquartile range). We compared them using Student *T* test or Mann–Whitney *U* test depending on normality of their distribution. The relationship between requirement for advanced respiratory support and selected variables (BQ score, STOP-BANG score, ESS score, NoSAS score, OSA-50 score, ODI, REI, minimal SpO_2_, time in bed spent with SpO_2_ < 90% (TIB90), hypoxic burden) was evaluated using separate logistic regression models with independent variables (age, sex, BMI, hypertension, diabetes mellitus) preselected based on the available literature and expert knowledge of the authors. This was a complete case analysis. A *p* value < 0.05 was considered significant. Statistical analyses were performed with R Studio, packages: dplyr and rms.

## Results

### Study group characteristics

Demographic and clinical characteristics of the study group can be found in Table 1.

**Table 1:** Baseline characteristics of the study group

Parameter	Value
Demographic data, measurements, and comorbidities	
Age, years	61 (49, 67)
Sex, male, *n* (%)	89 (71)
BMI, kg/m^2^	31.0 (26.9, 34.8)
Neck circumference, cm	41 (39–44)
Waist-hip ratio, cm	1.0 (0.9, 1.1)
Hypertension, *n* (%)	75 (60)
Chronic heart failure, *n* (%)	3 (2)
Coronary artery disease, *n* (%)	7 (6)
Stroke, *n* (%)	4 (3)
Diabetes mellitus,* n* (%)	32 (25)
Obesity, *n* (%)	66 (56)
Obstructive lung disease, *n* (%)	18 (24)
Questionnaires	
STOP-Bang, pts	3.5 (2, 5)
Berlin, pts	1 (1, 2)
ESS, pts	5 (2, 8)
NoSAS, pts	11 (9, 15)
Polygraphy	
REI	14.7 (7.2, 30.2)
OA	3.1 (1.2, 8.8)
H	8.9 (4.1, 17.5)
CA	0.3 (0.0, 0.8)
MA	0.4 (0.1, 1.1)
ODI	13.2 (6.1, 26.2)
SpO_2_ mean	91 (89, 92)
SpO_2_ minimum	81 (77, 84)
TIB90%, minutes	20 (2, 46)
Hypoxic burden, %min/h	21.5 (8.3, 50.6)
Maximal desaturation, %	8 (6, 11)
OSA severity, *n* (%) Mild Moderate Severe	103 (82) 41 (33) 30 (24) 32 (26)
Advanced respiratory support requirement	
HFNO, *n* (%)	81 (65)
PAP, *n* (%)	18 (15)

Data are presented as median (interquartile range) unless otherwise indicated. Abbreviations: *PAP*, positive airway pressure; *HFNO*, high flow nasal oxygen; *ODI*, oxygen desaturation index; *TIB90%*, time in bed in blood oxygen saturation below 90%; *H*, hypopnea; *MA*, mixed apnea; *CA*, central apnea; *OA*, obstructive apnea; *REI*, respiratory event index; *ESS*, Epworth Sleepiness Scale

Among enrolled 125 patients, the median age was 61 years (IQR, 49–67), and majority of patients were male. Patients tended to be obese with median BMI 31.0 (IQR 26.9, 34.8), waist-hip ratio 1.0 (IQR 0.9, 1.0), and neck circumference for women and men 38 (36–40) and 42 (40–45), respectively. The most frequently diagnosed comorbid conditions were hypertension, obesity, diabetes, and obstructive lung disease. A majority of patients (85%, 106/125) presented with a respiratory failure at admission, and the median MEWS score at admission was 1 (IQR 1–2).

Information on polygraphy results are presented in Table 1. Sensitivity, specificity, positive predictive value, and negative predictive value for ESS, BQ, STOP-BANG, NoSAS, and OSA-50 scores are presented in Table 2. Comparison of screening tools and polygraphy results stratified by OSA severity is presented in Supplementary Table 1.

**Table 2:** Accuracy measures for OSA screening tools

	Sensitivity	Specificity	PPV	NPV
STOP-BANG ≥ 3	66% (55–76%)	59% (36–79%)	86.3% (79–91%)	31% (22–42%)
Berlin questionnaire ≥ 2	55% (44–65%)	77% (55–92%)	90% (81 to 95%)	30% (24–38%)
NoSAS ≥ 8	71% (60–80%)	50% (28–72%)	85% (78–90%)	31% (21–43%)
OSA50 ≥ 5	78% (68–86%)	50% (28–72%)	86% (80–90%)	37% (25–51%)
ESS ≥ 10	18% (10–28%)	77% (55–92%)	75% (56–88%)	19% (16–24%)

### Outcomes

Advanced respiratory support was introduced in 85 patients (68%) and 8 (7%) patients eventually required intubation. All patients who required intubation were diagnosed with OSA. Ten patients (8%) required transfer to the ICU, and the median ICU stay was 10 days (9–13). Median hospitalization time was 16 days (11–26) and was longer in patients with OSA (18 vs. 11 days, *P* = 0.004). Taking into account OSA diagnosis and severity, there were no differences in frequency of HFNO use, while PAP was most frequently used in patients with severe OSA compared to patients with moderate OSA, mild OSA, and no OSA (31% vs. 10% vs. 8% vs. 9%, *P* = 0.02). Supplementary Table 1 presents more details.

**Fig. 1:**
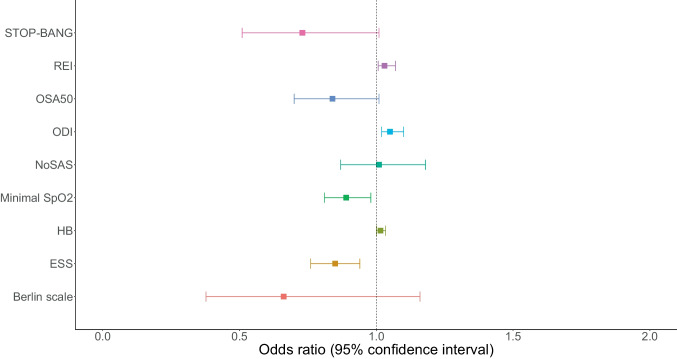
Summary of the multivariable analysis. Abbreviations: HB, hypoxic burden, ODI, oxygen desaturation index; REI, respiratory event index; ESS, Epworth Sleepiness Scale

Univariable comparison of patients requiring advanced respiratory support and the remaining patients is presented in Table 3. Multivariable analysis revealed that increased risk of requirement for advanced respiratory support was associated with higher REI (OR 1.03, 95%CI 1.00 to 1.07), higher ODI (OR 1.05, 95%CI 1.02 to 1.10), higher HB (1.02 95% CI 1.00 to 1.03), lower ESS score (OR 0.85, 95%CI 0.76 to 0.94), and lower minimal SpO_2_ (OR 0.89, 95%CI 0.81 to 0.98), but not with BQ score (OR 0.66, 95%CI 0.38 to 1.16), STOP-BANG score (OR 0.73, 95%CI 0.51 to 1.01), NoSAS score (OR 1.01, 95%CI 0.87 to 1.18), and OSA50 score (OR 0.84, 95%CI 0.70 to 1.01). The results of multivariable analysis are visualized in Fig. 1.

**Table 3:** Comparison of selected clinical characteristics between subgroups based on the occurrence of the primary outcome

Parameter	Primary outcome not achieved *N* = 40	Primary outcome achieved *N* = 85	*P* value
Demographic data, measurements, and comorbidities			
Age, years	62 (53, 69)	60 (47, 67)	0.245
Sex, male, n (%)	28 (70)	61 (72)	1.000
BMI, kg/m^2^	29.9 (26.9, 33.7)	31.6 (26.9, 35.1)	0.369
Neck circumference, cm	42 (38, 45)	41 (39, 44)	0.766
Waist-hip ratio, cm	1 (0.9, 1.1)	1 (0.9, 1.0)	0.454
Hypertension, *n* (%)	26 (65)	49 (58)	0.557
Chronic heart failure, *n* (%)	2 (5)	1 (1)	0.499
Coronary artery disease, *n* (%)	3 (8)	4 (5)	0.828
Stroke, *n* (%)	1 (3)	3 (4)	1.000
Diabetes mellitus, *n* (%)	10 (26)	22 (26)	1.000
Obesity, *n* (%)	18 (49)	48 (59)	0.380
Obstructive lung disease, *n* (%)	9 (23)	9 (11)	0.077
Age, years,	62 (53, 69)	60 (47, 67)	0.245
Questionnaires			
STOP-Bang, pts	4 (3, 5)	3 (2, 4)	0.036
Berlin, pts	2 (1, 2)	1 (1, 2)	0.230
ESS, pts	7 (5, 11)	4 (2, 7)	< 0.001
NoSAS, pts	12 (9, 15)	11 (9, 15)	0.578
Polygraphy			
REI	11.6 (4.8, 22.6)	15.9 (8.7, 31.4)	0.062
OA	3.1 (1.1, 8.8)	3.1 (1.2, 8.8)	0.709
CA	0.3 (0.0, 0.7)	0.3 (0.0, 0.8)	0.666
MA	0.4 (0.1, 1.1)	0.4 (0.1, 1.2)	0.856
H	6.1 (2.9, 12.1)	9.8 (5.0, 19.1)	0.025
ODI	11.5 (4.7, 20.0)	14.8 (7.3, 29.9)	0.013
SpO_2_ mean	92 (91, 93)	90 (89, 91)	< 0.001
SpO_2_ minimum	83 (80, 86)	81 (77, 83)	0.021
TIB90%, minutes	2 (0, 21)	30 (11, 54)	< 0.001
Maximal desaturation, %	7 (5, 9)	8 (6, 11)	0.051
Hypoxic burden, %min/h	11.3 (6.6, 38.1)	31.1 (11.6, 56.0)	0.076
OSA severity, *n* (%) Mild Moderate Severe	28 (70.0) 10 (25.0) 11 (27.5) 7 (17.5)	75 (88.2) 31 (36.5) 19 (22.4) 25 (29.4)	0.045

Data are presented as median (interquartile range) unless otherwise indicated. Abbreviations: *ODI*, oxygen desaturation index; *TIB90%*, time in bed in blood oxygen saturation below 90%; *H*, hypopnea; *MA*, mixed apnea; *CA*, central apnea; *OA*, obstructive apnea; *REI*, respiratory event index; *ESS*, Epworth Sleepiness Scale

## Discussion

Evidence on the prevalence of undiagnosed obstructive sleep apnea among patients with COVID-19 remains scarce. The current study revealed a very high rate of undiagnosed obstructive sleep apnea among patients hospitalized due to severe COVID-19. Furthermore, results from this single center study suggest that increasing severity of sleep-related breathing disorders is associated with higher requirement for more advanced respiratory support.

OSA is significantly underdiagnosed in many populations, and the current results are similar in order of magnitude to estimations made by Young et al. for the general US population [11]. The prevalence of undiagnosed OSA in our study group was very high and would very likely be even higher if patients who died were included in the analysis. According to available literature, the prevalence of previously diagnosed OSA in hospitalized patients with COVID-19 ranged from 15.3 to 20.9% [23, 24]. The frequency of sleep-related breathing disorders among COVID-19 patients in small prospective studies was assessed to be 62% and 79% among ARDS survivors [25, 26]. In a small study including 44 hospitalized COVID-19 patients, OSA was diagnosed in 75% of the study subjects [27]. In a German study on patients recovering from COVID-19, only 25.2% of the sample revealed no significant sleep apnea [28]. There are a few studies addressing OSA as a risk factor of COVID-19 morbidity and mortality. Some of them are based on retrospective analysis of medical records and are therefore biased  by the fact of undiagnosed OSA. Strausz S et al. found that patients with OSA had a 2.93-fold greater probability of COVID-19 hospitalization, independent of BMI and other established risk factors for OSA or severe COVID-19, implying that OSA is an independent risk factor for severe COVID-19 [24]. In a study by Maas et al. conducted in Switzerland, OSA was more prevalent among patients requiring hospitalization, and among those who progressed to respiratory failure, also after adjustment for diabetes, hypertension, and BMI [23]. Whereas Cade B et al. reported OSA patients to have increased all-cause mortality, yet loosing statistical significance when adjusted for BMI and OSA associated comorbidities [29]. Our findings show that number of respiratory events, measures of hypoxemia such as hypoxic burden, and number of desaturations as well as minimal saturation during the night are related to requirement for more advanced respiratory support. This is in line with previous research on COVID-19 and OSA, suggesting the importance of sleep-related breathing disorders in patient outcomes.

The association between OSA and worse outcomes in COVID-19 is multifactorial and encompasses, but is not limited to, below mentioned aspects. We hypothesize potential mechanism of how OSA may worsen the course of COVID-19. First, OSA leads to nocturnal desaturations during sleep, thus worsening the symptoms of acute respiratory failure in severe COVID-19. Thus, lack of positive airway pressure (PAP) delivered by HFNO or CPAP therapy may aggravate hypoxemia and result in anaerobic metabolism and its complications. Second, patients with OSA often have at least one comorbidity that is a known risk factor for severe COVID-19, e.g., obesity, hypertension, or diabetes. Third, OSA is related to increased inflammatory response, oxidative stress, and endothelial dysfunction that might add up to the damage caused by SARS-COV-2 infection. Early identification of patients who would benefit from PAP therapy seems to be essential of managing acute respiratory failure in the course of COVID-19.

In the current study, popular OSA screening tools had poor accuracy in identifying patients at increased risk of OSA. Interestingly, NoSAS and OSA-50 outperformed more widely used STOP-BANG score and BQ and presented highest sensitivity, yet not exceeding 80%. These questionnaires also failed to predict acute respiratory failure requiring advanced respiratory support in COVID-19 patients. STOP-BANG score sensitivity in predicting OSA among patients undergoing surgery was reported to be 83.6% in validation study, in contrast with the current study which was only 66.3% [30]. In previous studies, higher preoperative STOP-BANG scores were linked with postoperative ICU admissions, but it was also reported that the STOP-BANG score does not predict hypoxemia in adults recovering from non-cardiac surgery [31, 32]. However these screening tools were designed and validated in a different setting that is in cohorts of stable not hospitalized patients. Surprisingly, more severe respiratory failure was associated with on the one hand more severe sleep associated breathing disturbances and on the other less daytime sleepiness measured by ESS. In a study by Hayden MC et al., it was reported that patients who recovered from COVID-19 frequently were diagnosed with OSA, but with a negative association between symptoms of fatigue and AHI [28]. These consistent observations suggest a different than expected relationship between OSA severity and symptoms in patients who underwent COVID-19. Not without significance, subjective assessment of sleepiness and fatigue might have been influenced by time of inquiry. Previous research has suggested that not all patients with sleep apnea present with daytime sleepiness [33].

A strength of the current study was its prospective design, avoiding the bias of both undiagnosed disease and imprecise coding in medical records. There are limitations of this study. First, we have assessed the prevalence of undiagnosed OSA only in a selected population of patients requiring hospitalization in a single center study, thus limiting the generalizability of the results. Second, patients who died were excluded from the study, which could influence the results, probably by underestimating the number of patients with OSA. Third, the diagnosis was established during hospitalization due to COVID-19, when the patients no longer required oxygen support, yet very soon after recovery from acute phase of the disease. Fourth, the use of polygraphy, not polysomnography, may have lowered the precision of our estimates on OSA prevalence in the studied population.

## Conclusions

Previously undiagnosed OSA was common among hospitalized patients who survived the acute phase of COVID-19. The degree of OSA was related to the severity of respiratory failure.

### Supplementary Information

Below is the link to the electronic supplementary material.

Supplementary file1 (DOCX 17 KB)

## Data Availability

The datasets generated during and/or analyzed during the current study are available from the corresponding author on reasonable request.
