# Supplementary material for: Association between undiagnosed obstructive sleep apnea and severe course of COVID-19: a prospective observational study
Source: Sleep Breath. 2023 Jul 7;28(1):79–86. doi: 10.1007/s11325-023-02855-8 (PMC10954863; doi:10.1007/s11325-023-02855-8)
Supplement: Supplementary file 1 — Supplementary file1 (DOCX 17 KB) [file 11325_2023_2855_MOESM1_ESM.docx]

**SUPPLEMENTARY MATERIALS**

| **Supplementary Table 1.** Comparison of screening tool, polygraphy results and respiratory support between subgroups based on OSA severity. | | | | | |
| --- | --- | --- | --- | --- | --- |
| **Parameter** | **No OSA**  **N=22** | **Mild OSA**  **N=41** | **Moderate OSA**  **N=30** | **Severe OSA**  **N=32** | **P value** |
| **Questionnaires** | | | | | |
| STOP-Bang, pts | 2 (1, 4) | 3 (2, 4) | 4 (3, 5) | 5 (3, 6) | <0.001 |
| Berlin, pts | 1 (1, 1) | 1 (1, 2) | 1 (1, 2) | 2 (1, 2) | 0.029 |
| No-SAS, pts | 9 (7, 11) | 11 (7, 15) | 13 (11, 16) | 15 (11, 17) | 0.001 |
| ESS, pts | 5 (3, 9) | 5 (2, 8) | 4 (2, 7) | 6 (2, 8) | 0.946 |
| **Polygraphy** | | | | | |
| REI | 3.5 (2.5, 4.5) | 10 (7.3, 12.3) | 20 (17.3, 26.8) | 40 (34.3, 45.6) | <0.001 |
| OA | 0.4 (0.3, 1.1) | 1.5 (1.0, 4.4) | 5 (2.6, 7.8) | 14.8 (9.8, 20.4) | <0.001 |
| CA | 0.0 (0.0, 0.1) | 0.1 (0.0, 0.4) | 0.5 (0.0, 1.2) | 0.5 (0.3, 1.3) | <0.001 |
| MA | 0.1 (0.0, 0.1) | 0.2 (0.0, 0.6) | 0.5 (0.3, 1.1) | 1.4 (1.1, 2.2) | <0.001 |
| H | 2.5 (1.2, 3.2) | 5.4 (4.0, 9.1) | 12.9 (9.3, 18.3) | 20.5 (16.6, 25.8) | <0.001 |
| ODI | 3.1 (2.3, 5.3) | 7.4 (5.8, 11) | 19 (13.8, 24.5) | 32.5 (29.5, 35.3) | <0.001 |
| SpO_2_ mean, % | 93 (92, 93) | 91 (90, 93) | 90 (89, 91) | 90 (88, 91) | <0.001 |
| SpO_2_ minimum, % | 83 (82, 87) | 83 (80, 85) | 80 (76, 83) | 77 (73, 81) | <0.001 |
| TIB90%, minutes | 1 (0, 10) | 12 (1, 36) | 32 (12, 55) | 31 (15, 55) | <0.001 |
| Maximal desaturation, % | 6 (5, 6) | 7 (5, 8) | 9 (8, 11) | 11 (8, 13) | <0.001 |
| Hypoxic burden, %min/h | 6.3 (2.2, 9.4) | 12.8 (7.4, 21.3) | 35.7 (23.0, 59.9) | 59.4 (47.2, 93.8) | <0.001 |
| **Modality of respiratory support** | | | | | |
| HFNOT | 10 (45.5) | 30 (73.2) | 18 (60.0) | 23 (71.9) | 0.118 |
| PAP | 2 (9.1) | 3 (7.5) | 3 (10.0) | 10 (31.2) | 0.020 |
| HFNOT or PAP | 10 (45.5) | 31 (75.6) | 19 (63.3) | 25 (78.1) | 0.045 |
| IMV | 0 (0.0) | 3 (7.3) | 3 (10.7) | 2 (6.5) | 0.516 |

Data are presented as median (interquartile range) unless otherwise indicated. Abbreviations: ODI, oxygen desaturation index; TIB90%, time in bed in blood oxygen saturation below 90%; H, hypopnea; MA, mixed apnea; CA, central apnea; OA, obstructive apnea; REI, respiratory event index; ESS, Epworth Sleepiness Scale
